# Improving heart failure care with an Experience-Based Co-Design approach: what matters to persons with heart failure and their family members?

**DOI:** 10.1186/s12913-023-09306-w

**Published:** 2023-03-28

**Authors:** Anne-Marie Suutari, Johan Thor, Annika Nordin, Kristina Areskoug Josefsson

**Affiliations:** 1 The Jönköping Academy for Improvement of Health and Welfare, School of Health and Welfare, Jönköping University, Jönköping, Sweden; 2 Department of Internal Medicine and Geriatrics, the Highland Hospital (Höglandssjukhuset), Region Jönköping County Eksjö, Sweden; 3 The Department of Health Sciences, University West, Trollhättan, Sweden; 4 Department of Behavioral Science, Oslo Metropolitan University, Oslo, Norway

**Keywords:** Heart failure, Experience-Based Co-Design, Healthcare quality improvement, Patient and public involvement, Thematic analysis

## Abstract

**Background:**

Heart failure is a chronic heart condition. Persons with heart failure often have limited physical capability, cognitive impairments, and low health literacy. These challenges can be barriers to healthcare service co-design with family members and professionals. Experience-Based Co-Design is a participatory healthcare quality improvement approach drawing on patients’, family members’ and professionals’ experiences to improve healthcare. The overall aim of this study was to use Experience-Based Co-Design to identify experiences of heart failure and its care in a Swedish cardiac care setting, and to understand how these experiences can translate into heart failure care improvements for persons with heart failure and their families.

**Methods:**

A convenience sample of 17 persons with heart failure and four family members participated in this single case study as a part of an improvement initiative within cardiac care. In line with Experienced-Based Co-Design methodology, field notes from observations of healthcare consultations, individual interviews and meeting minutes from stakeholders’ feedback events, were used to gather participants’ experiences of heart failure and its care. Reflexive thematic analysis was used to develop themes from data.

**Results:**

Twelve service touchpoints, organized within five overarching themes emerged. The themes told a story about persons with heart failure and family members struggling in everyday life due to a poor quality of life, lack of support networks, and difficulties understanding and applying information about heart failure and its care. To be recognized by professionals was reported to be a key to good quality care. Opportunities to be involved in healthcare varied, Further, participants’ experiences translated into proposed changes to heart failure care such as improved information about heart failure, continuity of care, improved relations, and communication, and being invited to be involved in healthcare.

**Conclusions:**

Our study findings offer knowledge about experiences of life with heart failure and its care, translated into heart failure service touchpoints. Further research is warranted to explore how these touchpoints can be addressed to improve life and care for persons with heart failure and other chronic conditions.

**Supplementary Information:**

The online version contains supplementary material available at 10.1186/s12913-023-09306-w.

## Background

### Co-designing healthcare processes with persons living with heart failure

Heart failure (HF) is a chronic, and life-threatening heart condition typically caused by ischemic heart disease, hypertension, or valvular disease [1]. The prevalence of HF appears to be 1–2% of adults [1]. HF is a clinical syndrome with cardinal symptoms (e.g., breathlessness, ankle swelling, and fatigue) and typical signs (e.g., peripheral oedema). HF is “due to a structural and/or functional abnormality of the heart that results in elevated intracardiac pressures and/or inadequate cardiac output at rest and/or during exercise.” [1]. Clinical HF management guidelines promote acknowledging persons with heart failure (from now on referred to as PWHF) as equal partners in their healthcare, for example by engagement in monitoring and self-management of HF [1].

However, there are challenges to PWHF being equal partners to healthcare professionals. PWHF may experience a poor physical capability, making it difficult to cope with life, which can be a barrier to being engaged in healthcare services [2]. Furthermore, cognitive decline [3] and low health literacy (HL) among PWHF [1, 4–9], may make it difficult for them to understand and engage in healthcare service activities. In addition to HF-related problems, we found that additional barriers to being involved in healthcare service design include PWHFs’ and professionals’ limited knowledge about what co-production and co-design of healthcare processes means and how to apply these practices in real life healthcare contexts [2].

Co-production of healthcare processes with PWHF aims to promote the best possible health through joint learning about patients’, family members’ and healthcare professionals’ needs, circumstances, resources, and experiences [10–15]. Co-production and co-design can be conceptualized in a wide range of ways in healthcare and social contexts [16]. One cluster of definitions refers to Experience-Based Co-design (EBCD) [16]. EBCD is a step-by-step approach to healthcare quality improvement drawing on service users’ and professionals’ healthcare experiences [17, 18]. In this study, we wanted to test whether EBCD might be useful, as demonstrated in other patient groups, also when re-designing HF care with PWHF. This paper reports on the first steps of an EBCD initiative involving PWHF, their family members (who are sometimes also carers), and professionals in the re-design efforts.

### Experience-based Co-Design

EBCD is a participatory approach that draws on patients’, family members’ and professionals’ healthcare experiences [17, 18]. Stakeholders’ experiences are gathered through observations of healthcare consultations and individual interviews. The patients’ filmed or audio-recorded interviews are edited into a “trigger film” highlighting their care experiences and service touchpoints. Touchpoints are emotionally significant positive or negative events, situations or key resonating themes that shape patients’ overall service experience [19]. The “trigger film” prompts a patients’ workshop where a facilitator solicits their experiences to identify opportunities for improvement. Separate similar feedback events are held with family members and professionals to solicits their healthcare experiences and proposals for healthcare improvement. In a subsequent joint stakeholder workshop, patients, family members and professionals prioritize which healthcare service improvement opportunities to pursue further. Proposed service changes are then co-designed in small teams, each with patient, family member and professional representatives.

Results from earlier EBCD projects, e.g., in emergency care and mental health, indicate that EBCD can strengthen patients’ and family members’ voices, enable healthcare professionals to appreciate patients as equal partners in healthcare improvement efforts, and improve patients’ experiences of healthcare services [20–24].

### Experience-Based Co-Design with persons with heart failure

There are few research projects describing an EBCD process with PWHF. Raynor et al. [25] report on a researcher-initiated, adapted EBCD process including PWHF in both primary care and secondary care contexts. Raynor et al. aimed at testing the feasibility and acceptability of researchers co-leading EBCD as a part of intervention development. An intervention addressing PWHFs’ discharge from hospital, the “Medicines at Transitions Intervention”, was proposed. The intervention included, for example, education and information activities for and with PWHF, and intensified support from pharmacists, to improve PWHFs’ medication management. An EBCD project aiming to promote information and communication technology self-care support for PWHF found EBCD to be useful in the context of HF to discover the needs of PWHF and their family members regarding self-care [26]. Furthermore, EBCD improved professionals’ learning about the service users’ perspective.

### Knowledge gaps

**Fig. 1:**
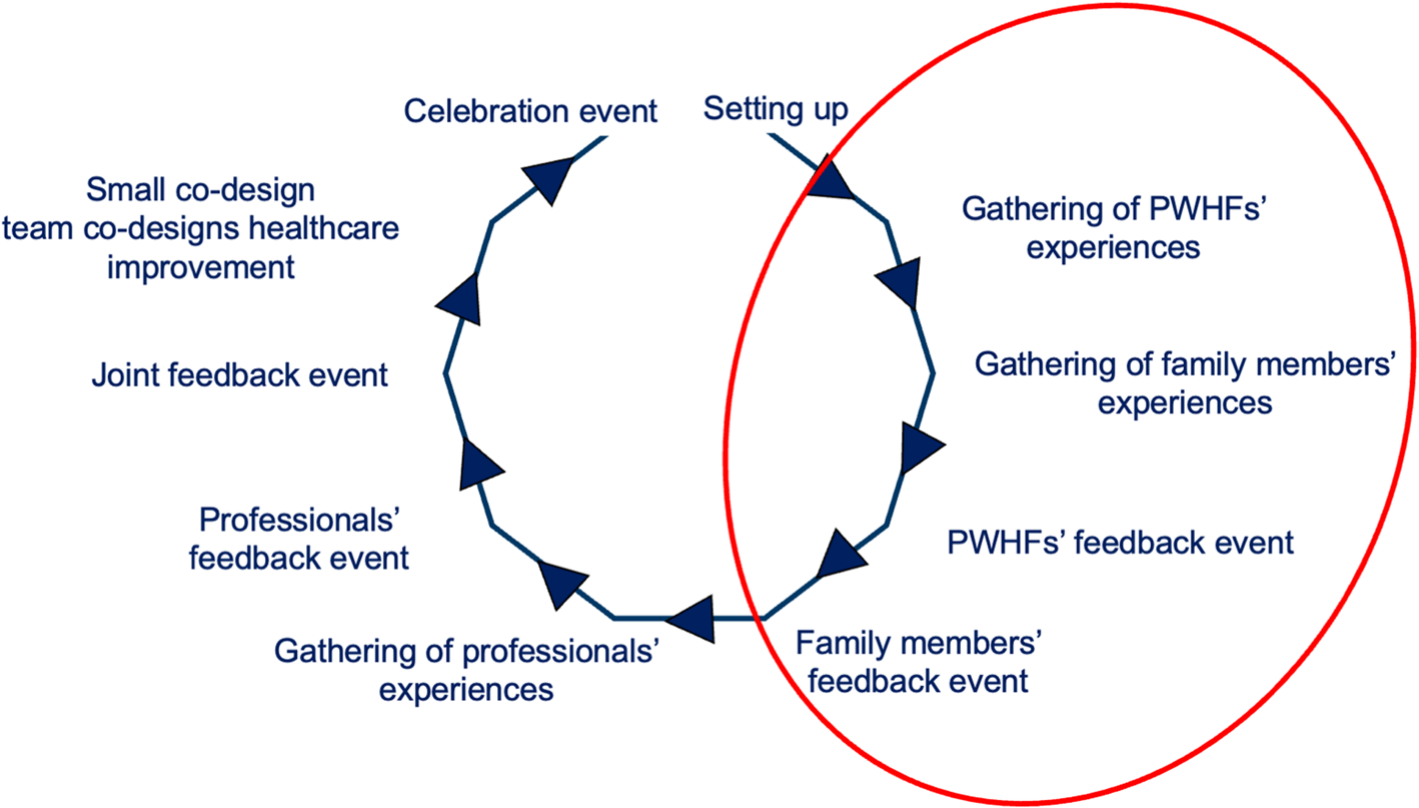
The EBCD process with the focus of this paper within the red circle (figure modified from Donetto et al., 2015 [27])

To our knowledge, this is the first study that has employed an EBCD approach in a Swedish cardiac care setting to explore PWHFs’ and family members’ overall experiences of life with HF and of HF care to inform the future co-design of HF care improvement. To date, there is limited knowledge regarding what experiences that can be identified among PWHF and their family members when using EBCD. Also, there is limited knowledge about what changes to HF care that are proposed based on identified stakeholders’ experiences. Our study adds to HF literature by using EBCD and stakeholders’ experiences to identify service touchpoints, useful to inform future improvements to HF care. This paper reports on the first steps of the EBCD approach – the gathering of PWHFs’ and family members’ experiences (Fig. 1). Professionals’ experiences, describing the co-design process and participants’ experiences of being involved in the EBCD process will be addressed in future studies.

### Aims

The overall aim of this study was to identify PWHFs’ and family members’ experiences of living with HF and of HF care in a Swedish cardiac care setting, and to understand how these experiences translate into proposed HF care changes. The research objectives are:

1. To identify experiences of life with HF and of HF care service touchpoints among PWHF and their family members.
2. To identify what changes to HF care PWHF and their family members propose based on the identified experiences and service touchpoints.

## Methodology

### Design, setting, participants, the EBCD process and data collection

This is a case study [28] of a single case EBCD initiative in a Swedish cardiac care setting in the southern parts of Sweden, conducted between October 2021 and May 2022. The EBCD process formed the first steps of an improvement initiative to improve HF care for PWHF and their family members.

EBCD adopts a pragmatic approach to participation and focuses on “what is useful”, thus supporting the involvement of those who want and can participate in healthcare quality improvement and research. A primary care nurse suggested eligible individuals for participation, then contacted by the main author for information about the process. Twenty one individuals (17 PWHF and four family members) formed a convenience sample after providing informed consent for study participation. Convenience sampling is a non-probability sampling strategy in which participants are selected for study participation if they are part of a target population and meet certain practical criteria [29].

Figure 1 offers a visual presentation of the EBCD process and the focus of our study. The EBCD process was guided by the Experience-based co-design toolkit [18]. First, PWHFs’ experiences were gathered through field notes from non-participant observations of their healthcare consultations. Next, filmed or audio-recorded individual interviews with PWHF solicited their experiences. The interviews were guided by a semi-structured interview guide developed by the authors to mirror different experiences of life with HF and of HF care, as well as proposed improvements to HF care (please see additional file 1 for the PWHF interview guide). These interviews were edited into a 30 min “trigger film” mirroring the PWHFs’ experiences. Next, family members’ experiences were gathered through semi-structured interviews guided by the same interview guide but focusing on their experiences of HF and its care. The following step, the PWHFs’ feedback event, aimed at validating the PWHFs’ experiences. The “trigger film” was used to prompt the discussions during this event. Preliminary key themes, developed during data analysis by the main author A-MS, were discussed with the participants during the event. A similar feedback event was held with family members to solicit their experiences.

After completing the gathering of the professionals’ experiences through individual interviews and a feedback event, a joint feedback event with all participating stakeholders was held. Again, the “trigger film” prompted the discussions. During the joint stakeholder event, participants jointly agreed on improvements to HF care. These improvements are then co-designed by small teams with patient, family member and professionals representatives. The process will end with a celebration event during which the joint work is celebrated and evaluated.

Online communication tools were used to facilitate data collection and stakeholder interaction during covid-19 pandemic meeting restrictions. The main author A-MS conducted the interviews and the first draft of the analysis. She had the role of a facilitator during the EBCD process. The participant recruitment process, steps taken in the EBCD process and data collection procedures have been reported in detail elsewhere by Suutari et al. (2022) [19].

### Participant roles

Co-production of healthcare services has evolved and grown over time in the context of this study [30]. However, structured involvement of persons living with disease, family members, healthcare professionals and citizens in service improvement and research is not yet routinely used within the study context [19]. Thus, this study offered an opportunity for stakeholders to overcome barriers to co-production and healthcare quality improvement.

PWHFs’ and family members’ research involvement in this study included [19]:

- Study participation: PWHF and family members participated in observations of healthcare consultations, individual interviews and EBCD feedback events.
- Research management: One PWHF representative and one family member representative participated in the steering group of the research project. These representatives co-planned and co-led the feedback events together with researcher A-MS and health professional representatives to ensure that all stakeholder perspectives were acknowledged throughout the process.
- Development of interview guides: To ensure clear interview questions, two PWHF and two family members reviewed the interview guides prior to the data collection.
- Data analysis: The identified service touchpoints and preliminary key themes were discussed with participants during separate stakeholder feedback events and during the joint feedback event to validate the analysis and to make sense of the findings.
- Future dissemination of research findings (to come): Participating PWHF and family members will be encouraged to be involved in joint study result presentations at seminars and conferences. They can also independently choose other contexts for presentation of the study results.

### Data analysis

Method triangulation, i.e., multiple research methods to collection data, was employed in our study. To address the research questions and to develop themes from collected data, inductive and latent reflexive thematic analysis (TA) was employed [31]. TA is a cluster of qualitative research methods aiming at producing meaningful knowledge [32]. This aligns with the pragmatic theoretical assumptions underpinning our study and data analysis. In this paper, pragmatism guided the data analysis to generate information that would be useful for improving HF care. Reflexivity means critically questioning one’s role in and impact on research [33]. In reflexive TA, researcher subjectivity is the primary tool for data analysis [34]. Subjectivity is considered to be something that is valuable and the key to successful reflexive TA, rather than something that is problematic. Thus, researchers should draw on previous experiences, knowledge and expertise when analyzing data.

Author A-MS is a practicing cardiologist in the study setting. Within her position, she interacts with PWHF and family members on a daily basis. This role gives her a preunderstanding, e.g., experiences and theoretical frames of reference, all of which are helpful when analyzing data [35]. A-MS was the lead researcher in this EBCD project, and thus is an insider researcher. All researchers, involved in this study, have expertise in quality improvement science, qualitative and case study research methods. The research group is interdisciplinary and covers experience of research and clinical work in cardiac care.

The analytical process followed the guidelines for reflexive TA [31] and development of themes with use of specific questions [36]. The study participants reflected on the content and meaning of the themes during EBCD feedback events. Further theme development included reflections from the researchers to tell a story about an important pattern around a central organizing concept related to the research questions. Thus, a form of investigator triangulation was used [37]. Aligning with the concept of inductive thematic saturation, saturation was achieved when no new codes och themes emerged [38].

## Results

### Participant characteristics

#### Persons with heart failure

##### Observations of healthcare consultations

Nine PWHF (three women and six men) participated in observations of healthcare consultations. No demographics, other than gender, were collected from these PWHF so as not to interfere with the consultations. One man, participating in observations of healthcare consultations, agreed to participate in subsequent steps of the EBCD process (PWHF7 in Table 1). The rest of the PWHF, who participated in observations, declined further study participation.

**Table 1:** Characteristics of PWHF participating in the interviews

Person with HF (PWHF)	Sex	Age, years	Years of HF diagnosis	Previous occupation	Participation, steps of EBCD process
PWHF1	Male	86	Several years^1^	Farmer	Individual interview
PWHF2	Female	83	4	Teacher, principal	Individual interview Joint feedback event
PWHF3	Male	68	1.5	Not applicable	Individual interview
PWHF4	Male	76	Several years^1^	Excavator operator, self-employed	Individual interview PWHF feedback event Joint feedback event
PWHF5	Female	85	2	Cemetery worker, maid	Individual interview PWHF feedback event Joint feedback event
PWHF6	Male	72	2	Construction industry	Observations Individual interview PWHF feedback event Joint feedback event
PWHF7	Male	76	9	Painter	Individual interview PWHF feedback event Joint feedback event
PWHF8	Female	82	0.5	Kitchen, daycare	Individual interview PWHF feedback event

^1^PWHF unable to tell

### Individual interviews

Eight PWHF (three women and five men, age range 68–86 years) participated in the interviews exploring experiences of living with HF, HF care and areas of service improvements. Five PWHF (two women and three men, age range 72–82 years) agreed to participate in a separate PWHF feedback event to validate the identified key themes. Five PWHF (two women, three men, 72–83 years) participated in the joint stakeholder event. Table 1 provides information about PWHF participating in individual interviews and subsequent EBCD steps.

### Family members

Four family members (four women, age range 46–71 years) participated in the interviews exploring experiences of living with HF, HF care and areas of service improvements. Three family members (three women, age range 46–69 years) agreed to participate in a separate family member feedback event to validate the identified key themes. Four family members (four women, age range 46–71 years) participated in the joint stakeholder event. Table 2 provides information about family members participating in individual interviews and subsequent EBCD steps.

**Table 2:** Characteristics of family members participating in the interviews

Family member (FM)	Sex	Age, years	Occupation	Relation to PWHF	Participation, steps of EBCD process
FM1	Female	46	Assistant nurse	Daughter	Individual interview Family member feedback event Joint feedback event
FM2	Female	69	Assistant nurse	Wife	Individual interview Family member feedback event Joint feedback event
FM3	Female	71	Assistant nurse	Wife	Individual interview Joint feedback event
FM4	Female	55	Assistant nurse	Wife	Individual interview Family member feedback event Joint feedback event

### Themes

Twelve service touchpoints were identified from the PWHFs’ and family members’ experiences gathered during the first steps of our EBCD process. Table 3 offers a summary of codes from observations and interviews, subthemes, service touchpoints and overarching themes developed from data. Five overarching themes emerged.

**Table 3:** Observations, codes from interviews, subthemes, service touchpoints and overarching themes

**Observations**	**Codes from interviews**	**Subtheme**	**Service touchpoints to be addressed during future HF**^a^ ** care improvements**	**Themes**
Various health issues	I have poor quality of life	PWHFs’^b^ experiences of living with HF	Lack of support when diagnosed with HF Lack of support networks to support PWHF	Theme 1: I struggle every day
	HF worries me but I try to cope			
	My family is my primary support system			
	HF worries me	Family members’ experiences of life with PWHF	Lack of support networks to support family members	
	My family is my primary support system			
Difficulties to understand heart failure and its treatment Healthcare professionals use medical language	I don’t understand HF	PWHFs’ experiences of health-related information	Inappropriate timing for information to PWHF Inadequate strategies for communication with PWHF	Theme 2: I don’t understand HF
	I need more information!			
	I don’t understand HF	Family members’ experiences of health-related information	Inappropriate timing for information to family members Inadequate strategies for communication with family members	
	I need more information!			
Satisfied patient Professionals are in charge	I am treated in a good way	PWHFs’ experiences of relations with professionals	Patient-professional interactions: lack of acknowledgement Lack of care continuity	Theme 3: Please, do not ignore me!
	I have confidence in the professionals			
	Please, listen to me!			
	Please, let me see my physician!			
	Please, listen to me!	Family members’ experiences of relations with professionals	Family member-professional interactions: lack of acknowledgement	
	Please, let my family member see his/hers physician!			
Engaged PWHF Professionals are in charge Engaged family member	Motivation to be involved	PWHFs’ involvement in healthcare processes	Professionals do not invite PWHF to be involved in healthcare	Theme 4: How can I get involved?
	Capability to be involved			
	Opportunity be involved			
	Motivation to be involved	Family members’ involvement in healthcare processes	Professionals do not invite family members to be involved in healthcare	
	I am not sure when and how to be involved			Theme 5: HF care can improve!
	Information about HF and its care			
	Relations with professionals			
	Continuity of care			

^a^*HF * Heart failure, ^b^*PWHF * Persons with heart failure

Theme 1, “I struggle every day”, focuses on the everyday struggles of PWHF and their family members, highlighting a poor quality of life and inadequate support systems. Theme 2, “I don’t understand HF” focuses on the difficulties in obtaining, understanding, and applying health-related information. Theme 3, “Please, do not ignore me!” focuses on PWHFs’ and family members’ experiences of not being listened to during consultations. Theme 4, “How can I get involved?”, focuses on PWHFs’ and family members’ motivation, capabilities, and motivation to be involved in healthcare processes. Theme 5 “HF care can improve!”, includes stakeholders’ proposed improvements to HF care based on their experiences.

#### Theme 1: I struggle everyday

##### PWHFs’ experiences of living with HF

Participating PWHF recognized their multimorbidity and talked about other medical conditions sometimes being more disabling than HF itself. PWHF experienced that HF, combined with its underlying conditions, highly impaired quality of life. Shortness of breath and the lack of physical strength, which pushed individuals to contact healthcare, was described as having a particularly negative impact on quality of life:

"Now it's over. Now I can't do too much, I get so out of breath as soon as I move, you know. “(PWHF1)

To avoid shortness of breath, PWHF are instructed to take diuretics, i.e., “water pills” when noticing a worsening of symptoms. Diuretics help rid the body of salt and water through increased diuresis (increased secretion of urine). Diuretics, alongside other drugs for HF treatment, were experienced by PWHF to be “a necessary evil”. Although diuretics were recognized to ease symptoms of excess body fluid, they simultaneously can reduce the quality of life seriously:

"If you have taken them [the diuretics], you are tied for 6 -7 hours before it [the diuretic effect] wears off.” (PWHF1)

Being diagnosed with HF was experienced to be a life changing event by most participating PWHF. Reactions to being diagnosed with a life-threatening disease varied from not caring at all to feeling relieved to finally understand what was wrong. However, PWHF were constantly worried and anxious due to HF:

" I guess I thought that now I have to be prepared to die any day. I won't have any stamina." (PWHF3)

The loss of physical strength combined with worries about their future further impaired PWHFs’ quality of life. Analysis of interviews with PWHF identified various strategies for coping with HF. Overall, PWHF tried to keep a positive attitude toward life and live normal lives despite HF. One PWHF revealed that he tried to suppress that he was sick. Another PWHF found comfort in trying to understand his condition, reading everything about HF he came across. Trying not to worry was yet another coping strategy employed by one of the participating women.

To understand PWHFs’ support systems, they were encouraged to describe who they turned to for support during tough times. The closest family members, i.e., the spouse or children, were recognized to be the main ones they turned to. However, PWHF said that they wanted to keep their closest family members out. One reason for this was the idea of being a burden to their family if complaining too much about their health condition:

"I don’t want to put any burdens on my daughter because I want to try to ease things for her instead if possible. And my husband and my sister, no, we do not talk about illnesses.” (PWHF5)

No PWHF talked about looking for emotional support from healthcare professionals. One woman explained during the PWHF feedback event that physicians and nurses were probably more interested in medical stuff than in PWHFs’ experiences. However, PWHF missed the support from others living with HF and called for forums where experiences of life with HF could be shared.

##### Family members’ experiences of life with PWHF

Family members recognized HF to be a life-threatening disease. Noticing a decline in their loved ones’ physical capability and overall health condition was scary and concerning to family members. Worry was described as ever-present in their daily life:

“You get hypersensitive when you are worried, you sleep poorly at night, and you listen to his breathing.” (Family member (FM)2)

Emergencies, during which family members had feared for the life of their loved ones, were particularly stressful. Still, years after an emergency, a wife panicked when seeing or hearing an ambulance, recalling a traumatic event in the past.

A difficult aspect of family members’ life was trying to create a balance between being worried and trying to put on a brave face for their loved ones. Rather than talking about their worries and anxiety with their sick family member, they offered them emotional and practical support in everyday life. To cope, most family members turned to healthy family members for support and said they were reluctant to seek support from people outside the closest family circle. Only one family member mentioned having friends that she was able to talk to about her worries. In line with participating PWHF, no family member mentioned looking for emotional support from healthcare professionals.

#### Theme 2: I don’t understand HF

##### PWHFs’ experiences of health-related information

PWHF were found to have difficulties understanding their medical condition. Observations during healthcare consultations revealed that health-related information seemed difficult to get across to some PWHF, leaving them unsure of treatment and next steps. Not being able to absorb and retain health-related information during healthcare visits can be a barrier to understanding HF:

"They have probably tried to give me information, but I have not been able to take it in […] I do not really understand what it [heart failure] means." (PWHF5)

Observations revealed another barrier to understanding health-related information by revealing that healthcare professionals tended to use medical language instead of plain language when communicating with PWHF.

Whereas some PWHF recognized having difficulties to understand and apply the information given to them, others spoke about not getting enough HF-related information from healthcare professionals. PWHF talked about professionals’ use of medial language bring a barrier to understanding information about HF. PWHF reasoned that healthcare professionals do not always recognize their needs for information. PWHF said they lacked information about HF, underlying causes, and symptoms, while others felt that they needed more information about HF treatment. One man explained that he suffered from post-traumatic stress after a life-threatening medical emergency that was not explained to him during or after the event. Thus, feeling uninformed can leave PWHF feeling scared and unsafe for a long time. Rather than blaming healthcare professionals for not providing them with enough information, PWHF wanted to take responsibility for asking for more information and for looking up information online and in books and brochures.

##### Family members’ experiences of health-related information

Family members, in line with PWHF, also spoke about sometimes having difficulties understanding information about HF. Not being able to understand what was happening during emergencies, was a particular source of anxiety and stress. In certain situations, family members felt excluded and not a priority to healthcare professionals, leaving them with a sense of being uninformed. To obtain information about what was said during healthcare consultations was not easy either, since their family members were not capable of recalling what had been said or just did not want to keep their family members informed:

“My husband doesn’t tell me everything, but I can see that he is not doing well.” (FM2)

The lack of information about how to self-manage HF was particularly unsettling to family members. Wanting to support PWHF to cope with HF, they felt unsure of what to do to prevent worsening of HF. When worsening of HF was a fact, PWHF with low health literacy often needed help to contact healthcare. However, family members lacked clear information about who to contact and when, making them feel further insecure and stressed out. “Why not just provide us with a phone number so that we know who we can contact?” family members reasoned. This implies that complex healthcare organizations are inaccessible and hard to navigate.

All family members in this study happened to be healthcare professionals. Thus, their experiences of life with a PWHF and their experiences of HF care probably differ from those of family members who do not work in healthcare. Nevertheless, one of the participating spouses testified that even though she had worked as an assistant nurse, her feelings, and her capability to handle HF, were not the same when her husband developed HF, compared to when she worked with HF patients. This implies that, although family members might be capable of finding information about HF for themselves, they may have difficulty in understanding – or managing – how this information applies to their loved ones.

#### Theme 3: Please, do not ignore me!

##### PWHFs’ experiences of relations with professionals

Overall, participating PWHF were satisfied with how they were treated by healthcare professionals. Professionals were considered to be pleasant and accommodating. PWHF had great confidence in the professionals who they had met during healthcare consultations and who they described as knowledgeable and easy to interact with. To be able to have a dialog with the professionals, rather than one-way communication, was important for the overall experience of care. Being able to trust the professionals made PWHF feel safe:

"You feel so well taken care of […] All the patients must love her [the primary care nurse]! She has something special; I was euphoric when I left.” (PWHF5)

However, less satisfactory situations were also highlighted during interviews. PWHF had experienced not being listened to during healthcare visits. “You are not allowed to have your own opinion”, one PWHF said remembering a situation during a healthcare visit. The non-participant observations of healthcare consultations also included situations where professionals did not always draw on PWHFs’ experiences, thoughts and issues when diagnosing and treating disease. Participating PWHF thought that not being listened to was due to professionals feeling overwhelmed and stressed out, thus not having the time to listen to their patients. PWHF reasoned that another explanation for when patients – or family members – were not listened to, was that, perhaps, some professionals wanted to have the upper hand during healthcare visits. Lack of care continuity, forcing PWHF to recapitulate their medical history to stranger professionals and having difficulties building trust, were also considered to have a negative impact on interactions between patients and professionals. Not being listened to made PWHF feel unsafe, stressed and not welcome to contact care:

"I made a visit here [to the primary care center] because I felt very sick... And then I got another doctor who almost scolded me for coming here and disturbing him." (PWHF6)

##### Family members’ experiences of relations with professionals

In line with PWHF, family members had experienced encounters when professionals did not listen to them. Family members felt that they constantly had to stand up for themselves and their sick family members to get sufficient attention from the professionals. Not being taken seriously and feeling ignored frustrated them. PWHF may well have difficulties explaining their health issues during healthcare consultations, some family members said. If professionals did not listen to them, no-one would know about the situation at home, and severe consequences for patient safety may arise, family members reasoned.

A lack of care continuity was experienced to be a barrier to building trustful relationships with healthcare professionals—having to constantly rebuild relationships all over again made the family members feel insecure:

“How you are treated is so important in healthcare, and that you have a team around you, and you should not have to change physicians and nurses all the time because it is so hard.” (FM4)

#### Theme 4: How can I get involved?

##### PWHFs’ healthcare involvement

Observations of healthcare consultations revealed that some PWHF were capable of engaging in their own care. These individuals asked questions and showed that they were knowledgeable about HF, which helped them to self-manage HF. However, interviews revealed that not all PWHF considered themselves to be capable to be involved in healthcare. The reasons for not being involved varied. Some PWHF just were not interested in being involved at all. Others thought that their experiences would not be interesting or important enough to inform care improvements. Yet others explained that they did not find it appropriate to tell professionals how to run healthcare:

“I am not the kind of person who wants to intrude. You accept things as they are.” (PWHF8)

##### Family members’ healthcare involvement

Observations of healthcare consultations and interviews indicated that family members felt highly motivated to be involved in the care of their loved ones. A wife of a PWHF said that she had been present at all her husband’s healthcare visits to support him. Another wife kept track of how her husband was doing and helped him to contact the doctor's office if needed. Others asked medical questions and helped the sick family member to explain his or her concerns during healthcare consultations. However, although family members were motivated to be involved, they felt unsure about when and how. Family members talked about their loved ones not wanting to involve them and not informing them about what was said during healthcare visits. Thus, family members found it difficult to be involved in a balanced way, i.e., to be involved without being too protective or interfering with the integrity of their loved one.

Observations of healthcare consultations showed how professionals usually were in charge during consultations, and did not always listen to the family member’s perspective:

“They [the physicians] do not look at me, they just look at the sick one […] I am supposed to just be quiet there, in the background.” (FM1)

Not being explicitly invited by professionals to be involved in the care of their loved ones was challenging to family members. “Why not just invite us and let us be a natural part of healthcare teams?” one family member reasoned. If professionals value the help from family members, maybe PWHF also would let us in to be involved, family members said. Wanting to help and support but feeling unsure about how and when to be involved represented a difficult balancing act among family members.

#### Theme 5: HF care can improve!

PWHF and family members suggested a range of improvements to HF care, drawing on their experiences (Table 4).

**Table 4:** PWHFs’ and family members’ proposed improvements to HF care

PWHFs’ **suggested improvements**	**Family members’ suggested improvements**
Improved information about HF •Symptoms •Treatment (medications and physical activity) •Timing for information about HF	Improved information about HF •Self-management of HF •Who to contact and when? •Timing for information about HF
Improved continuity in care	Improved continuity in care
Improved relations and communication	Improved relations and communication
	Invitation to be involved in healthcare

All PWHF and family members suggested improvements to information about HF, indicating that this was an important issue for all participants. First, *what* are the facts that need to be included in information about HF? PWHF talked about wanting to know more about HF symptoms, treatment, and physical activity to treat HF and to prevent HF from worsening. To relieve anxiety during emergencies, oral information about HF and its treatment should be clear and give hope for the future. Family members talked about wanting to know more about self-management of HF. Family members mentioned having stressful experiences of not knowing who to contact and when, in situations when their loved one’s health condition worsened. Thus, they suggested improvements to information about how, when and who to contact.

Second, *how* did PWHF and family members want to obtain information? PWHF and family members asked for both written and oral information about HF. PWHF wanted access to written information in plain language – to have something to go back to after healthcare consultations. Both PWHF and family members also suggested group activities with others in a similar situation, for the opportunity to exchange experiences about life with HF, for support and for co-learning about HF. One PWHF explained why he thought group activities would be a good idea:

“It might be a little easier to open up if you are with others who have the same medical condition. If you are the only one living with a disease, then you do not really talk about it.” (PWHF7)

Information communicated during emergencies was particularly hard to understand according to participants. Therefore, the *timing*, i.e., when health-related information is provided, was experienced to influence the understanding of HF and its care. Optimally, information should be repeated during planned healthcare visits, participants said.

Both PWHF and family members experienced that poor care continuity had a negative impact on care experiences and relations with professionals. Thus, they suggested that the continuity of HF care should improve, i.e., that they would be able to meet the same physician or nurse at subsequent healthcare visits. To be treated well and to be taken seriously by professionals, and to improve communication during healthcare visits were other improvements suggested by both PWHF and professionals. This was suggested to be accomplished by providing professionals with communication training. Since family members felt unsure about how and when to be involved in healthcare, they wished professionals would clearly invite them to be involved in healthcare, for example by including this request in invitations to healthcare consultations.

## Discussion

### Principal findings

In this paper, patient and public involvement refers to co-design, with persons living with disease, family members and professionals collaboratively re-designing healthcare services together, drawing on stakeholders’ service experiences [16, 17]. To our knowledge this is the first study that has employed an EBCD approach to explore PWHFs’ and family members’ overall experiences of life with HF and of HF care. Five themes, reflecting PWHFs’ and family members’ experiences and suggestions, emerged from field notes on healthcare consultations, interviews, and meeting minutes from PWHFs’ and family members’ feedback events.

The first theme, “I struggle everyday”, revealed PWHFs’ physical and emotional struggles in life with HF. Anxiety, a heavy burden of symptoms and side effects from medicines added to a poor quality of life. Both PWHF and their family members lacked support networks outside the closest family circle, which sometimes made it hard to cope with life with HF. The next theme, “I don’t understand HF”, revealed PWHF had health literacy challenges, i.e., difficulties in understanding and applying health-related information. Timing, context, and communication strategies were found to influence how the information provided was perceived and used. Feeling uninformed left PWHF feeling scared and unsafe. Family members lacked information about self-management strategies and who to contact and when, leaving them feeling insecure about how to support their sick loved ones. The third theme, “Please, do not ignore me!” focused on the quality of relations between PWHF, family members and professionals. Being recognized and listened to was highly important for how care was experienced. The fourth theme, “How can I get involved?” revealed that PWHFs’ and family members’ motivation, capabilities, and opportunities to be involved in healthcare varied. Not all PWHF wanted to be more involved in their own care or in healthcare processes and some reasoned that they did not have anything to offer. Family members were highly motivated to be involved, but felt unsure of how and when, and thus wanted to be explicitly invited to participate. The last theme, “HF care can improve!" drew on the PWHFs’ and family members’ experiences and suggestions for improvements to HF care. Their improvement areas included i) individualized information about HF and its treatment and ii) improved relations, communication, and iii) continuity of care. Family members highlighted their willingness and desire to be invited and to participate in healthcare.

### Comparison with prior work

#### Poor quality of life has a negative impact on everyday life

Quality of life can be defined as “an individual’s perception of their position in life in the context of the culture and value systems in which they live and in relation to their goals, expectations, standards and concerns” [39]. Our findings suggest that PWHF have a poor quality of life due to a heavy burden of HF symptoms, anxiety, and comorbidities. These findings are in line with other research studies with non-EBCD designs suggesting that PWHF have moderate to poor quality of life [6, 40–42].

Several factors explain poor health-related quality of life among PWHF. First, according to a European review, age and sex predict quality of life, with older female PWHF experiencing worse quality of life compared to younger male PWHF [43]. Second, depression, caused by HF symptoms such as shortness of breath and low physical energy, is associated with reduced quality of life among PWHF [44]. Although no PWHF in our study mentioned feeling depressed, all participating PWHF talked about being worried and anxious. Research suggests that PWHF have a high prevalence of anxiety and of depression, and that there is a risk of these conditions going unnoticed and undiagnosed, further worsening quality of life [43, 45, 46]. Third, low health literacy levels among PWHF seem to be associated with poor quality of life [47]. Not understanding HF and its treatment and not being able to apply this information to everyday management of HF, probably explain the association between health literacy and quality of life.

Emotional distress among family members when witnessing a partner or a parent becoming critically ill is not uncommon [48]. Family members in our study reported feeling distressed in everyday life and lacking support networks to be able to cope with HF in everyday life. These findings are in line with Grant & Graven [49] and Grigorovich et al. [50], who reported that caregivers’ access to social support predicts their emotional outcomes. Access to social support is associated with positive emotional outcomes whereas depressive symptoms among carers are associated with less involvement in PWHFs’ care. Thus, healthcare should be organized not only to provide medical assistance but also to provide emotional support to those in need.

#### Low health literacy makes it difficult to navigate in healthcare settings

PWHF in our study said that they had difficulties in obtaining, retaining, understanding, and applying health-related information. Several aspects, relevant when seeking to improve HF care, influence PWHFs’ experiences of health-related information. First, the timing and the context of information provision matter. The first author’s clinical experiences indicate that PWHF, who typically are older and sometimes suffering from cognitive impairment, need continuous and repeated information during all stages of the disease. Second, PWHF with low HL have difficulties in understanding, applying, and asking for information. These PWHF challenges need to be recognized by healthcare professionals so they can tailor information to fit the needs of each person living with disease. Third, how information is provided matters. Knowledge about how to employ different communication strategies to address PWHFs’ needs should be included in healthcare professionals’ training [51]. Strategies to help patients ask questions, should be developed, and tested [52].

These findings suggest that PWHF may have low HL. HL includes (a) knowledge of health and healthcare; (b) the ability to apply health-related information; and [3] the ability to self-manage disease and partner with healthcare professionals to improve one’s health [53]. Among PWHF, 20–50% have been reported to have low HL [6, 8, 54]. Predictors of low HL include high age, few years of education, cognitive function, and a high rate of coexisting illnesses such as diabetes, hypertension, chronic obstructive pulmonary disease, and stroke [8, 54]. PWHF in our study had a mean age of 78.5 years, were not highly educated, and lived with comorbidities, thus presenting with several characteristics predicting low HL. In addition, sometimes PWHF struggle to understand health-related information because the professionals do not use plain language when talking to them. Research in an Iranian context [55], revealed that professionals may occasionally withhold important information from PWHF, for example information about HF prognosis, thus preventing them from seeing the whole picture. Clear explanations about HF were sometimes not provided due to the patients’ mental and emotional conditions. Whether these findings can be translated into European and Swedish healthcare contexts remains uncertain.

Low HL among PWHF has several important consequences. First, low HL is associated with a poor quality of life [47]. Second, it is associated with a high all-cause mortality and a high risk of hospitalizations and visits to the emergency department – undesirable both for patients and to health systems [54, 56]. Third, low HL may make it difficult to engage in HF self-management activities [57–59], which in turn may explain why PWHF are at risk of HF exacerbations leading to hospitalizations [60]. Having support from family members or caregivers promotes self-management behaviors among PWHF [61]. However, some of the family members in our study talked about difficulties in understanding and applying health-related information, making them struggle to fully support their sick loved ones. Previous research indicates that low HL levels among carers are associated with poor self-management abilities among PWHF [62].

#### Good relations matter to overall service experience

Patient-reported complaints in healthcare settings frequently include dissatisfaction with communication between patients and professionals [63], not being met in a professional or caring way [64, 65] and not experiencing trust in healthcare professionals [46]. These findings suggest that the way you are treated, i.e., if you are acknowledged and listened to during healthcare consultations, matters to the overall experience of healthcare services, which was also evident in our findings. The findings mirror those in other EBCD projects. An EBCD project in a mental health care context recently indicated that patients and family members do want to be acknowledged and validated in healthcare [66]. Another EBCD project in a cancer care context revealed similarly that communication and relationships between stakeholders mattered for the overall experiences of care [67]. This implies that the quality of interactions between patients and professionals is crucial to good quality care. Thus, healthcare organizations should not strive for excellence only in adhering to guideline-recommended disease management but also when it comes to the quality of human interactions.

#### Co-designing healthcare

The motivation, capabilities, and opportunities to be involved in healthcare varied among participating PWHF in our study. These study findings are in line with our previous study on the usefulness of the Capability, Opportunity, and Motivation Behavior (COM-B) model [68] when assessing the barriers to and facilitators of co-production [2]. Some PWHF displayed low self-efficacy thinking – that they would have nothing to offer when co-designing healthcare processes and did not want to interfere with the professionals’ domains. In contrast, participating family members were highly motivated to be involved in healthcare processes. However, our findings indicate that family members sometimes feel excluded from the care of their loved ones. Both their loved ones and healthcare professionals seemed to exclude family members from participation in healthcare processes. Similarly, Wingham et al. [48] reported that some caregivers were excluded from consultations by PWHF, who refused to talk about their current situation or future plans. Some caregivers felt they were ignored by healthcare professionals during consultations, and thus were excluded from being involved in healthcare processes, which was similar to our findings [48].

The study findings imply that the PWHF and family members in our study felt excluded from healthcare involvement. This indicates that an uneven power balance between service users and professionals may exist. In our previous research study on co-production of healthcare services in a Swedish HF care context, participants spoke of a rather traditional “doctor-knows-best” healthcare context, with professionals being in charge of consultations [9]. Pirinen, exploring the barriers and enablers to service co-design [69], reported that it can be difficult “to persuade doctors to commit to a co-design project where the benefits were uncertain. It was difficult to introduce new roles to the strong professional power hierarchy in healthcare. Hierarchic relations affected the social dynamics and willingness to bring forth problems in the co-design sessions”. These findings resonate with Nimmon & Stenfors-Hayes [70] who found that physicians perceive themselves to hold power when interacting with their patients. Although some participating physicians found it natural to empower their patients, others experienced that their power weakened when patients were involved in care processes [70]. Thus, uneven power dynamics among patients, family members and professionals still seem to be a barrier to the co-design of healthcare processes. Employing a critical reflective practice and dialog [70–72] has been suggested to promote an awareness of professionals’ power and to balance power dynamics in healthcare. There is a risk, however, that these approaches are not prioritized in busy healthcare contexts with time and resource constraints.

#### Proposed improvements to HF care

There are several similarities between the study findings from our study in an HF care context and previous EBCD projects in various healthcare contexts, for example in mental health care [21, 66] palliative care [24] and cancer care [67]. Within the mental health care context [66], patients and carers suggested streamlining working practices to free up time for interaction with clinicians, more patient-led training for nurses on how to address their problems and taking a non-judgmental approach to patients and carers. Patients and family members within a palliative care context [24], proposed improvements on how they are acknowledged and validated by healthcare professionals. These findings are in line with our findings, with participants wanting to improve relations between stakeholders in the HF care setting. Within the cancer care context [67], participants wanted to improve communication between professionals and patients. Persons with breast cancer proposed that patients should be given the option to receive health-related information in groups with others. Persons with lung cancer suggested that patient information leaflets for specific points in healthcare processes should be produced [67]. Likewise, PWHF in our study expressed their need for written information about HF and its treatment whereas family members wanted to know more about self-management strategies. Similar to PWHF and family members in our study, persons with lung cancer expressed their frustration with changing care providers often and stated their desire for improved continuity of care [67]. Thus, our study findings validate the findings from other EBCD projects.

#### Methodological considerations

This study included a convenience sample of PWHF and family members. Convenience sampling is a sampling strategy in which participants are selected for study participation if they are part of a target population and meet certain practical criteria [29]. Advantages of convenience sampling are that it is cheap and simple to implement. Since those who want to participate are included, dropout is rarely an issue. A major disadvantage is the lack of clear generalizability [29]. However, being pragmatic about involving frail individuals in research, a convenience sampling strategy seemed appropriate for our study. EBCD adopts a pragmatic approach to participation and focuses on “what is useful” and supports the involvement of those who want and can participate in research [73]. Small sample sizes are further common during, as in our case, the first steps of a healthcare quality improvement initiative [73].

Only four family members were recruited for this study as family members were particularly difficult to engage. Previous research suggests that recruitment through trusted caregiver agencies is the most effective mechanism for family member recruitment for co-design research, although this sampling strategy may not result in a representative sample [74]. In our study, a primary care nurse suggested eligible family members for participation. This sampling strategy proved to be time intensive since many family members needed to be asked to find those who were interested in study participation. The recruited family members were all female healthcare professionals, which probably made the sample non-representative. Other recruitment strategies may therefore be needed for future co-design projects.

Having an embedded researcher perform the data collection and lead the project has pros and cons. There may be a risk of social desirability [75], meaning that the cardiologist and the researcher roles may influence who chooses to participate in the study or what information participants are willing to share during interviews and feedback events. PWHF and family members might feel obliged to participate due to fear of otherwise not getting access to healthcare when needed. To minimize this risk, the PWHF treated by the lead researcher were excluded from participation. Participants shared both good and negative HF care experiences, which indicated a safe environment for sharing lived experiences. Also, most participants agreed to participate in consecutive steps of the EBCD process, indicating that the process was perceived to be interesting and worthwhile.

## Conclusions

Our study findings offer knowledge about experiences of life with heart failure and its care, translated into heart failure service touchpoints. Further research is warranted to explore how these touchpoints can be addressed to improve life and care for persons with heart failure and other chronic conditions.

### Implications for practice

Our study findings have several implications for practice. First, the findings expand the understanding of life with a chronic disease. It needs to be recognized that PWHF and their family members may experience physical and emotional distress in their everyday life due to disease. This distress may limit their ability to fully engage in care decisions, in co-design of healthcare processes and in research projects. Therefore, they need to participate on their own terms. To facilitate participation, practical and emotional support should be available for patients and family members.

Second, low HL among PWHF and their family members must be recognized by healthcare professionals and researchers. Adapted communication strategies should be employed to help individuals to understand treatment options and diagnostic workups during healthcare consultations, thereby being able to be involved in shared decision-making. Similarly, adapted communication strategies need to be employed in research projects involving individuals with low HL. Clear communication about research objectives and what study participation involves may facilitate the recruitment of individuals otherwise underrepresented in research.

Third, uneven power relations need to be managed to facilitate “true” co-designed healthcare processes in which patients, family members and professionals work side by side with equally shared power. We propose that healthcare professionals discuss and reflect upon power issues that may arise in patient-clinician interactions. In the context of co-designed research, power issues should be discussed among participants prior to research participation and reflected upon during the co-designed process to highlight power issues.

Fourth, we propose that improvements to HF care, suggested by PWHF and family members participating in our study, may also be useful when aiming to improve care for other groups of individuals living with chronic conditions. Thus, we hope the identified areas of improvement can be helpful for other organizations when prioritizing where to start improving care.

## Supplementary Information

**Additional file 1.**

## Data Availability

Research Data at the School of Health and Welfare is regulated under the Freedom of the Press Act (1949:105) and the Public Access to Information and Security Act (2009:4) as Public Records/Official Documents. Research data that is registered and archived at the School of Health and Welfare can be requested anonymously by anyone for a fixed fee according to the Fee Regulation (1992:191). If the research data contain sensitive information, such as personal data and/or trade secrets, that information is protected by Confidentiality and Secrecy by the Public Access to Information and Security Act, and requests for such information will be denied with the possibility to appeal the denial in the Court of Appeals. Secret or confidential research data can be accessed by other researchers if they receive permission from the Regional Ethics Review Board. The datasets used and/or analyzed during the current study are available from the corresponding author on reasonable request. The lawful basis for transmission of secret and/or confidential information is then based on the Law of Ethics Review for Research on Humans (2003:460).

## Abbreviations

EBCD: Experience-Based Co-Design
HL: Health literacy
HF: Heart failure
PWHF: Persons with heart failure
TA: Thematic analysis
