# Supplementary material for: Improving heart failure care with an Experience-Based Co-Design approach: what matters to persons with heart failure and their family members?
Source: BMC Health Serv Res. 2023 Mar 28;23:294. doi: 10.1186/s12913-023-09306-w (PMC10044106; doi:10.1186/s12913-023-09306-w)
Supplement: Supplementary file 1 — Additional file 1. [file 12913_2023_9306_MOESM1_ESM.docx]

**Interview guide - Individual interviews with persons living with heart failure**

This interview guide guides the individual interviews with persons living with heart failure. The purpose of the interview is to identify your experiences of living with heart failure and its care. These experiences are important to identify "touch points" (health and care-related events and situations that created a strong positive/negative feeling). These "touch points" can then in turn be used to identify which areas within heart failure care needs to improve.

Question areas that will be touched upon during the interview are:

- From diagnosis to follow-up
- Overall satisfaction with heart failure care, the information you received, the influence you had and support.
- What has worked well and what needs to improve?

You do not need to have experiences from all these areas to be able to participate in the interview.

**Area 1: From diagnosis to follow-up**

**1.1 The first time you felt something was not right**

- When were you diagnosed with heart failure?
- How did you notice that something was wrong?
- What was your first reaction? What went through your mind?
- What happened then?

**1.2 The first healthcare visit (due to heart failure)**

- Where did you have your first healthcare visit? At the primary care center or at an emergency room?
- What happened during your first healthcare visit?
- What information did you receive?
- What was important to you during these visits?
- How did you feel?
- What were your first impression of the care facility?
- What memories do you have of the first meeting with the healthcare professionals?

**1.3 Later healthcare visits**

- What happened? How did it feel?
- What stood out to you during these visits? What was particularly important to you?

**1.4 Diagnostic work-up**

- What medical examinations have you done?
- Tell me about the time when you were waiting for the medical examinations.
- How did you experience the examinations?
- Tell me about the time when you were waiting for the results from your examinations.

**1.5 Your visits to the cardiac outpatient clinic**

- Have you visited the cardiac outpatient clinic at the hospital?
- For how long did you wait before you got an appointment at the cardiac outpatient clinic? How did you experience this wait?
- What are your memories of that first visit?
- What are your memories of the first meeting with your cardiologist?
- What are your memories of your first meeting with the nurses and other staff?
- What are your memories of later visits to the cardiac outpatient clinic?

**1.6 Family and friends**

- What role have your family and friends played during your time of sickness?
- What was their response? (Problematic? Supportive?)

**1.7 Treatment**

- Tell me about the time when you started treatment due to heart failure.
- How did you experience your hospital admission?
- How did you feel when you were discharged from the hospital?

**1.8 Follow-up**

- Tell me about your follow-up visits.

**Area 2: Overall satisfaction with heart failure care, the information you received, the influence you had and support.**

**2.1 Overall satisfaction with heart failure care**

- How satisfied have you been so far with the care and treatment you have received?
- What are the best bits and worst bits?

**2.2 The information you received**

- How have you experienced the communication between you and the professionals?
- What information have you received about heart failure and its treatment?
- What would you have liked more information about? Why?

**2.3 The influence you had**

- In what ways have you been able to influence your own care?
- Are there any things in which you would like to have had more ‘say’?

**2.4 Your relations with the healthcare professionals**

- Tell me about your relationships with the healthcare professionals. How do/did you experience this relationship?
- What could the professionals have done differently?

**2.5 Your support system**

- How have you experienced your support system?

**2.6 How did you manage to cope?**

- What advice would you give to other people about how to develop a personal coping strategy and how to stay in control of your own life?

**Area 3: What has worked well what can improve?**

- What were the best and worst parts of your whole experience?
- Where would you say are the crucial points in the journey – moments of truth?
- What parts of the healthcare service do you think that we need to focus on to be able to improve heart failure care?
- Based on your experiences, if you were looking to redesign and improve the services for heart failure patients, where would you begin? Imagine we were setting it all up from scratch.

**Would you like to add anything else?**

**THANK YOU!**
